# Biomechanical behavior of three maxillary expanders in cleft lip and palate: a finite element study

**DOI:** 10.1590/1807-3107bor-2024.vol38.0010

**Published:** 2024-04-08

**Authors:** Angela Maria Bautista Patiño, Monise de Paula Rodrigues, Roberto Sales Pessoa, Salomón Yezioro Rubinsky, Ki Beom Kim, Carlos José Soares, Guilherme de Araújo Almeida

**Affiliations:** (a) Universidade Federal de Uberlândia – UFU, School of Dentistry, Department of Pediatric Dentistry and Orthodontics, Uberlândia, MG, Brazil.; (b) Universidade Federal de Uberlândia – UFU, School of Dentistry, Department of Operative Dentistry and Dental Materials, Uberlândia, MG, Brazil.; (c) Universidade Federal de Uberlândia – UFU, School of Dentistry, Uberlândia, MG, Brazil.; (d) Universidad Nacional de Colombia, School of Dentistry, Department of Orthodontics, Bogotá, Colombia.; (e) Saint Louis University, Center for Advanced Dental Education, Department of Orthodontics, Saint Louis, MI, USA.

**Keywords:** Finite Element Analysis, Palatal Expansion Technique, Bone Screws, Cleft Palate

## Abstract

This study evaluated the stress distribution in the dentoalveolar and palatal bone structures during maxillary expansion in a 17-year-old male patient with bilateral cleft lip and palate (BCLP) using expanders with dental (HYRAX) and skeletal anchorage (MARPE). For the generation of the specific finite element models, cone-beam computed tomography was used, and the DICOM files were exported to Mimics 3-Matic (Materialise) and Patran (MSC Software) software. Three specific three-dimensional models were generated: A) HYRAX: conventional four-banded hyrax screw (9 mm); B) MARPE-DS: 3 miniscrews (1.8 mm diameter – 5.4 mm length) and four-banded dental anchorage; and C) MARPE-NoDS: 3 miniscrews without dental anchorage. Maxillary expansion was simulated by activating the expanders transversely 1 mm on the "X" axis. HYRAX resulted in higher levels of deformation predominantly in the dentoalveolar region. MARPE-DS showed stress in the dentoalveolar region and mainly in the center of the palatal region, at approximately 4,000 με. MARPE-NoDS exhibited evident stress only in the palatal region. High stress levels in the root anchoring teeth were observed for HYRAX and MARPE-DS. In contrast, MARPE-NoDS cause stress on the tooth structure. The stress distribution from the expanders used in the BLCP showed asymmetric expansive behavior. During the initial activation phase of expansion, the HYRAX and MARPE-DS models produced similarly high strain at the dentoalveolar structures and upper posterior teeth displacement. The MARPE-NoDS model showed restricted strain on the palate.

## Introduction

Cleft lip and palate (CLP) is considered the most common craniofacial anomaly in humans.^1,2^ These malformations involve the upper lip, alveolar ridge, and palate.^3^ In general, CLP causes esthetic, functional, and psychosocial impacts at different magnitudes, depending on its location and extension.^3^

Treatment normally starts in early childhood and involves maxillary expansion (ME) before the secondary alveolar bone graft procedure.^4–6^ The ME protocol is a well-established method for correcting transverse maxillary deficiency in children due to skeletal maxillary expansion through the opening of the midpalatal suture with few undesirable dental effects.^7,8^ However, in late-adolescent and adult patients, nonsurgical palatal expansion can result in uncontrolled dentoalveolar tipping, unfavorable periodontal effects,^9^ root resorption,^10^ and a high relapse rate of orthodontic treatment due to skeletal resistance.

Recently, the use of temporary skeletal anchorage devices with ME has resulted in a decrease in the side effects of conventional maxillary expansion by achieving pure skeletal changes.^11^ The miniscrew-assisted palatal expander (MARPE) is a simple modification of a conventional ME appliance. The main difference is the incorporation of miniscrews into the palatal jackscrew to ensure the expansion of the underlying basal bone and minimize dentoalveolar tipping.^11,12^

The effects of ME treatment have been extensively studied over time using different methods, including three-dimensional finite element analysis (FEA).^9,13,14^ FEA is an important method for evaluating the biomechanical effect of a complex structure by dividing the complex domain into a finite number of interconnected elements,^15,16^ which allows the visualization of the displacements and deformation, which is distributed by color scales.^17^ When the bone, periodontal ligament, and tooth structures are subjected to a load, then stress, strain, and displacement are resultants. The magnitude of the load, the design of the orthodontic devices, and the anchorage method can determine the resultant displacement, strain, and stress.

The stress distribution in ME by HYRAX, tooth-borne or tissue- and bone-borne, and bone-borne palatal expanders have been extensively studied.^9,11,13,14,17–21^ They have demonstrated that different designs, especially bone-borne expanders, presented distinct stress distributions.^18,19^ Compared with bone-borne appliances, HYRAX and tooth bone-borne expanders can result in a dentoalveolar buccal inclination as a side effect.^18–20^ On the other hand, the arms between the bone-borne screw and the upper posterior teeth used as anchorage sometimes have been considered determinants to stabilize the expander.^21^ The extension and location of the cleft palate may prevent one or more screws from being positioned, suggesting possible instability of the expander unless the arms between the upper posterior teeth and the screw are installed. The dentoalveolar and skeletal effects of MARPE and conventional expanders using only skeletal anchorage or associated with dental anchorage for ME for treating cleft lip and palate are not clear and need further studies.

The aim of this FEA study was to analyze the displacement, stress, and strain distribution in the dentoalveolar and maxillary palatal structures resulting from ME in a complete bilateral CLP using two different types of bone-borne expanders (MARPE, with or without dental anchorage) and conventional tooth-borne palatal (HYRAX) expanders by a 3D finite specific patient model.

## Methodology

This study was approved by the ethical committee (at the National University of Colombia and Pediatric Hospital *La Misericordia* protocol B. CIEFO-243-18). A cone-beam computed tomography image was selected from the tomographic image bank of the Orthodontic Clinic of the Pediatric Hospital *La Misericordia*, Bogotá, DC, Colombia.

A maxillary scan of a 17-year-old teenager with bilateral full cleft lip and palate who received a successful secondary bone graft (cancellous iliac crest bone) at age nine, before the eruption of the permanent canine, was used in this study. This patient had complete permanent dentition from the second molars, with the absence of the right central and lateral incisors, and both canines and first premolars erupted in transposition.

A structural nonlinear three-dimensional FEA was created from a cone-beam computed tomography scan (CS9300 Carestream, 90 kV, 15 mA, field of view 10x5 cm, 0.18 mm slice thickness and 0.3 mm voxel dimension) using Mimics software (version 18.0; Materialise, Leuven, Belgium).

**Figure 1:**
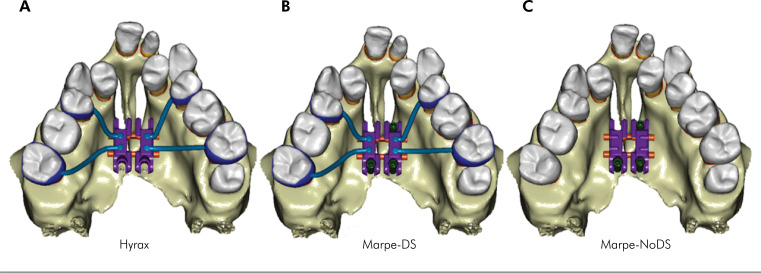
Specific models by finite element analysis. A) HYRAX; B) MARPE-DS; C) MARPE-NoDS.

Segmentation of the maxillary bone was performed from the incisal edge of the teeth to the zygomatic bone height. The different structures, compact bone, cancellous bone, enamel, and dentin, were accomplished using image density thresholding.^22,23^ A periodontal ligament layer 0.2 mm thick,^24^ was imposed by Boolean operations.^22^ After segmentation, the 3D triangle-based surface of each maxillary structure was exported in stereolithography (STL) format. The orthodontic bands and connecting arms to the expander were designed using 3-Matic software (version 18.0; Materialise, Leuven, Belgium). The STL surface files were imported and meshed in MSC. Patran® 2010 (MSC. Software, Santa Ana, USA) with tetrahedral elements was used to form a volumetric element mesh. This mesh was imported into an FEA software package (MSC. Marc/Mentat, MSC. Software) to perform the structural analysis. Tetrahedral elements with 134 element types were used for both the bone and miniscrew systems to ensure smooth contact at the interfaces. Nodes on top of the bone structure, except for palatal bone, were rigidly fixed in the x-(horizontal), y-(vertical), and z-directions. The top of the maxillary bone was also fixed. All materials were considered linear-elastic, isotropic, and homogeneous. The applied material properties (elastic modulus and Poisson's ratio) were obtained from the literature (Table 1).^9,25–29^ Interfaces between the structures were considered bonded to prevent motion, which means that the tooth structures and bone were not allowed to separate, also preventing the rotation of the maxillary section. The expander screw contact was considered rigid. Three models were generated (Figure 1):

**Table 1:** Material properties and elements used in the present study.

Structure	Elastic Modulus (MPa)	Poisson's ratio	References
Enamel	84 100	0.30	21
Dentin	18 600	0.31	22
Periodontal ligament	50	0.45	23
Trabecular bone	1370	0.30	24
Cortical bone	13700	0.30	24
Miniscrew (titanium)	110 000	0.30	25
Expander and Band (Stainless steel)	200 000	0.30	9

*HYRAX model*: Conventional hyrax screw with 9 mm (PecLab Ltda, Belo Horizonte - MG, Brazil) and four bands (both upper second premolar and second molar);

*MARPE-DS model:* MARPE SL 9.0 mm with dental anchorage (PecLab); dental four-banded anchorage (both upper second premolar and second molar) supported by 3 miniscrews (PecLab) with a diameter of 1.8 mm and a length of 5.4 mm, which were placed lateral to the midpalatal area;

*MARPE-NoDS model:* MARPE SL 9 mm without dental anchorage (PecLab) supported by 3 miniscrews (PecLab) with a diameter of 1.8 mm and a length of 5.4 mm, which were placed lateral to the midpalatal area.

In the HYRAX and MARPE-DS models, the left and right second premolars and second molars were banded. The bands were meshed using shell elements connected to teeth using a bonded interface and connected with 1.5 mm stainless steel wire to the base of the expander screw and the lingual surface of the bands on both sides.

Each model consisted of 1.286.756 elements and 5.502.525 nodes, and the total data processing time was 120 hours per model. Expanders were activated transversely by 0.1 mm for 10 steps, resulting in a total of 1.0 mm of expansion in the *X* direction and were unfixed in the *Y* and *Z* directions to prevent interference with the resultant movement. The displacement (mm), von Mises stress (MPa), and equivalent elastic strain (με) distributions were assessed at the dentoalveolar bone, maxillary palatal bone and anchorage teeth.

## Results

**Figure 2:**
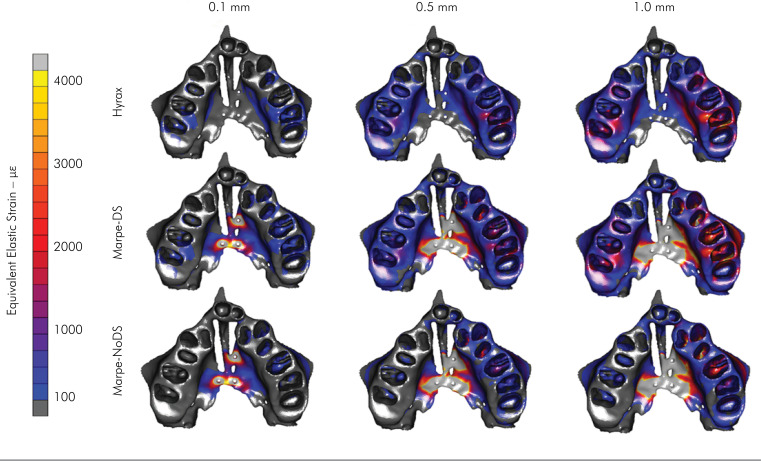
Stress distribution (*μ*ε) in the alveolar and palatal bone structures, based on the three expanders used.

The strain distributions (με) in the bone structure for the 3 orthodontic devices are shown in Figure 2. The HYRAX model resulted in concentrated strain in the dentoalveolar bone. However, the resultant strain on the palatal bone was null. The MARPE–DS model resulted in the highest strain, *i.e.*, approximately 4.000 με at the midpalatal region. MARPE–DS resulted in dentoalveolar bone strain similar to that of HYRAX. MARPE-NoDS had the highest strain concentrated in the palatal region. Null strain was observed at the dentoalveolar bottom region.

**Figure 3:**
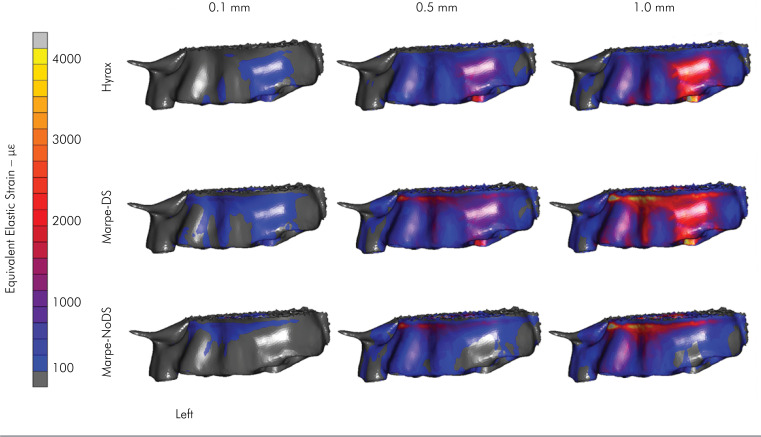
Left side of the strain distribution in the buccal alveolar bone surface.

**Figure 4:**
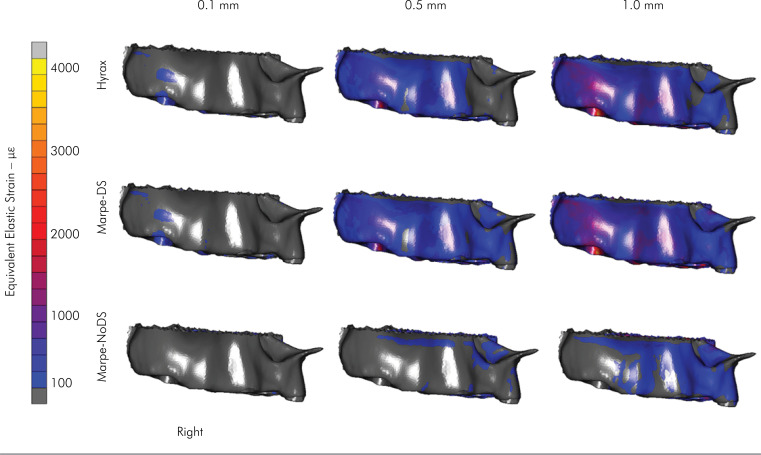
Right side of the strain distribution in the buccal alveolar bone surface.

The strain distributions on the left and right sides of the buccal alveolar bone are shown in Figures 3 and 4, respectively. On the left side, the HYRAX device resulted in a strain concentration peak (≅2.000 με) around the molar region when simulating 1 mm of expansion. The MARPE-DS expander showed a similar strain distribution to the HYRAX expander and a high strain concentration at the palate bone. The MARPE-NoDS expander showed predominant strain distribution at the palatal bone and practically null strain concentration on the posterior teeth (Figure 3). On the right side, the HYRAX and MARPE-DS devices showed similar strain concentrations at the buccal maxilla bone, with lower strain (≅1.000 με) than at the left side (Figure 4). This behavior was observed with the MARPE-NoDS expander, where the strain concentration was minimal on the whole buccal right side. When comparing the strain concentration between the left and right sides, it was evident that this distribution was asymmetric (Figures 3 and 4).

**Figure 5:**
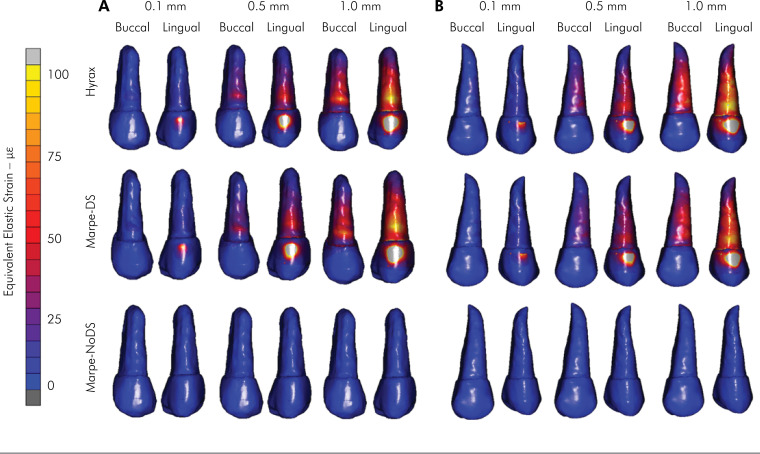
Von Mises stress distribution on the buccal and lingual surfaces of the right (A) and left (B) upper second premolars.

**Figure 6:**
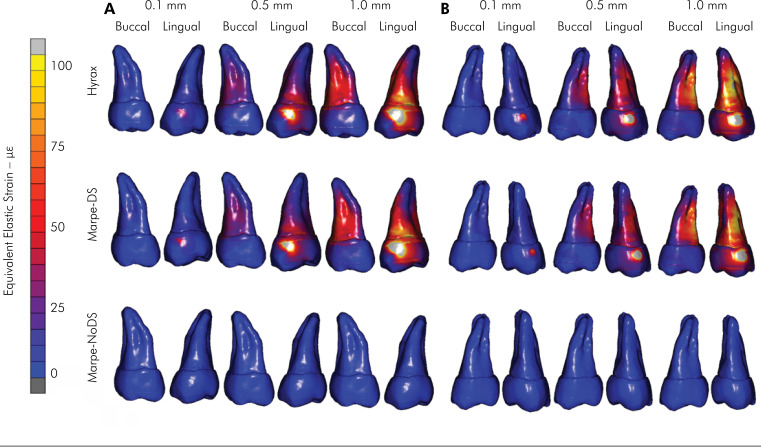
Von Mises stress distribution on the buccal and lingual surfaces of the right (A) and left (B) upper second molars.

The von Mises stress distributions on the buccal and lingual surfaces of the premolar and molar teeth used to stabilize the orthodontic devices are shown in Figures 5 and 6 and in Table 2. HYRAX resulted in the highest stress concentration in the cervical dentin region. The left and right premolars showed similarly high stress levels (≅100 MPa). The left second molar had higher stress (≅115 MPa) than the right second molar (Table 2). The MARPE-DS expander demonstrated a similar stress distribution to that of the HYRAX device on posterior teeth. The MARPE-NoDS model showed low stress concentrated on posterior teeth (Figures 5 and 6) (Table 2).

**Table 2:** Maximum von Mises stress distribution and displacement in the dentin of molar and premolar teeth where orthodontic devices have been stabilized.

Devices	Equivalent von Mises Stress (MPa)				Displacement (mm)			
	Premolar		Molar		Premolar		Molar	
	Left	Right	Left	Right	Left	Right	Left	Right
Hyrax	103.7	101.2	114.3	86.6	0.23	0.24	0.32	0.2
Marpe-DS	89.3	100	108.3	86.2	0.25	0.24	0.33	0.2
Marpe-NoDS	6.2	0.4	6.2	1.1	0.05	0.00	0.03	0.00

Considering the tooth displacements, the left second molar showed slightly greater displacement than the right second molar when HYRAX and MARPE-DS were used. The displacement of the upper second premolars was similar for the two expanders. With the MARPE-NoDS device, the second premolars and molars had practically no displacement (Table 2).

## Discussion

In this study, a maxillary expansion simulation of 1.0 mm was adopted since this amount of activation would be enough to generate a significant deformation of the 3D elements.

The strain distribution in the distal palatal region and the displacement of the involved teeth were different according to the ME and the type of anchorage used. In the occlusal view, the HYRAX expander, with the use of a strictly dental anchorage, showed a higher strain at the dentoalveolar region (3200 με) and a null effect on the palatal location. On the other hand, for MARPE-DS and MARPE-NoDS, which use skeletal anchorage expanders, the highest strain distribution occurred around the miniscrews on the palatal bone (Figure 2). These findings are confirmed by previous studies that demonstrated that the concentration of strain in the palate is very similar for expanders with skeletal anchorage.^9,14^ The use of skeletal anchorage during the initial phases of ME in patients with cleft lip and palate generated a high strain concentration (> 4000 με) in the central region of the palate, which was able to induce bone cell activity in this area. Thus, the skeletal effect of MARPE-type expanders has been demonstrated in several clinical trials.^6,19,20^

This study showed that even with MARPE-DS presenting skeletal anchorage, the dentoalveolar strain was very similar to the dentoalveolar results yielded by HYRAX.^18–20^ In contrast, the MARPE-NoDS expander had no significant strain in the posterior teeth, limiting its strain concentration to the palate bone (Figure 2 and Table 2).^18–20^ The difference between HYRAX and MARPE-DS with MARPE-NoDS performance can be explained by the force generated in the initial expansion that is transferred by the arms to the teeth.^18–20^ On the other hand, regardless of the type of expander, the strain tended to be more concentrated around the screws and teeth involved in the anchorage and smaller at the anterior maxilla bone (Figure 2). That region was likely influenced by the more posterior positioning of both expanders due to the presence of palate clefts. In this particular case, it was necessary to use the second premolars and molars for anchorage, and it was not possible to install a fourth miniscrew in the left anterior region of the MARPE-DS and MARPE-NoDS expanders due to the extension of the palate gap in this patient.

The lateral strain distributions in the dentoalveolar and palate regions maintained the same behavior as those in the occlusal view, according to the type of anchorage (Figures 3 and 4). The expansion was asymmetric, with a greater strain concentration on the left side than on the right side. This asymmetry can be explained by two factors. First, the cleft width determines lower bone strength when the cleft size is greater. In this study, the most extensive cleft palate was located on the right side. Second, the asymmetry is related to the number of screws used. In the MARPE-DS and MARPE-NoDS expanders, there was one more screw on the left side due to the impossibility of installing a second screw on the right side because of the greater extension of the cleft palate in this region. Consequently, substantial resistance to the movement of the dentoskeletal structures was imposed on the left side, generating greater strain concentrations. This became clear due to the presence of strain distribution only in the region of the palate when MARPE-DS and MARPE-NoDS expanders were used. For these expanders, the intensity of the deformation found on the palate was directly related to the number of screws used and the bone strength present in each posterior segment, according to the cleft palate extension (Figures 3 and 4, Table 2). This finding was consistent with previous studies^12,30,31^ that demonstrated that the stress distribution between the cleft and noncleft sides was asymmetric because of differences in the masses and support structures of the minor and major segments of the maxilla.

The HYRAX and MARPE-DS expanders resulted in the highest strain around the buccal bone plate, especially in the left second molar area. The presence of arms connected to dental structures (MARPE-DS) generated a high concentration of strain in the dentoalveolar structures of the buccal bone plate, even when associated with skeletal anchorage. These results support the clinical finding when evaluating the periodontal effect in different types of maxillary expanders.^8,18–20,32^

The stress distribution in the posterior teeth was dependent on the dental anchorage. When a MARPE-NoDS-type expander was used, no displacement was observed because the anchorage of the expander occurred strictly on the skeletal bone tissue. On the other hand, when HYRAX and MARPE-DS were used, a significant stress level was observed in the second premolars and upper molars due to posterior tooth anchorage. The stress distributions were not uniform, manifesting in decreasing order from the crown to the root and on the buccal and lingual surfaces, with a greater concentration on the lingual surface, due to the arms connected to the expansion screw proximity. The left side showed the highest stress concentration, probably due to the lower resistance of the midpalatal suture to displacement, producing the expansion resultant transmitted to the tooth structures.^30^ Although the von Mises stress distribution method is not able to distinguish the type of stress, whether from compression or tensile stress, this finding also confirms the previous observations about the questioned use of conventional HYRAX expanders in patients with CLP when high buccal tipping is preexisting.^33^ According to this study, it seems logical to consider that in the presence of noncarious cervical lesions, short roots, periodontal disease, and posterior teeth with excessive vestibular inclination, MARPE-NoDS should be the expander of choice.^19^ On the other hand, given the relative integrity of the dentoalveolar structures of the posterosuperior segments, the choice between HYRAX and MARPE-DS would involve considering the patient's age and whether there is a need for predominantly skeletal expansion.^18–20^

However, in patients with cleft palate, this reasoning does not always apply clinically. First, there is a possible limitation in the number of screws to be installed, depending on the extent of the palate cleft, which can compromise the stability of the MARPE-NoDS expander and force the need to insert double arms between the screw and a possible dental anchorage.^21^ Additionally, in cleft palates, the gingival tissue present in the palatine region tends to be thicker, preventing bicortical insertion of screws, requiring a length of the transmucosal part of the miniscrew that is usually not available, which limits its potential for skeletal anchorage (especially with the MARPE-NoDS expander) and favors the option for HYRAX.^21^

Several factors, such as the shape of the palate and other anatomical structures, cleft width, and bone density, can affect the biomechanical system of maxillary expansion in patients with BCLP, making it difficult for only one FEA model to represent all clinical situations. Therefore, future clinical studies are recommended to investigate the effects of bone expanders on maxillary expansion in patients of different ages, types of cleft palate, and periodontal conditions, and involving all craniomaxillary structures.

## Conclusions

Based on this study, the following conclusions can be drawn:

- The distribution of stresses from the expanders used in the BLCP showed asymmetric expansive behavior.
- During the initial activation phase of expansion, the Hyrax and MARPE-DS expanders produced similarly high strain at the dentoalveolar structures and upper posterior teeth displacement.
- The MARPE-NoDS expander showed restricted strain on the palate.
